# Apical Sealing Ability of Bioceramic Paste and Mineral Trioxide Aggregate Retrofillings: A Dye Leakage Study

**Published:** 2015-03-18

**Authors:** Shuang Shi, Dan-Dan Zhang, Xu Chen, Zhi-Fan Bao, Ya-Juan Guo

**Affiliations:** a *Department of Pediatric Dentistry, School of Stomatology, China Medical University, Shenyang, China; *; b *Key Laboratory of Oral Disease of Liaoning Province, Shenyang, China; *; c * Department of Stomatology, General Hospital of PLA, Beijing, China*

**Keywords:** Bioceramic, iRoot FS, Mineral Trioxide Aggregate, Root-end Filling, Sealing Ability

## Introduction

**Introduction:** This study compared the apical sealing ability of a bioceramic based root canal repair filling material (iRoot FS) with mineral trioxide aggregate (MTA). **Methods and materials:** Forty single-canal teeth were prepared and randomly divided into two experimental groups (*n*=18) and two control groups (*n*=2). Teeth in experimental groups were retrofilled with either MTA or iRoot FS. After setting of the retrofillings, all roots were exposed to 0.2% Rhodamine B solution for 48 h and were then washed for 12 h before longitudinal sectioning. The extent of dye penetration was measured under fluorescence microscope. **Results:** The mean leakage values in MTA and iRoot FS samples were 35.63 and 35.15 µm, respectively. There was no significant differences between the two materials in this regard (*P*=0.584). **Conclusion:** According to dye leakage results, iRoot FS had similar apical sealing ability to MTA and might be considered as a promising root-end filling material.

## Introduction

When nonsurgical root canal treatment proves unsuccessful or contraindicated, surgical endodontic therapy is indicated to preserve the tooth. The procedure includes exposure of the involved apex, resection of the apical end of the root, preparation of a retrograde cavity inside the canal and finally filling the cavity with a root-end filling material [1]. Apical sealing is the most important factor in successful periapical surgery [2]. The apical sealing is provided by the root-end filling material and can prevent re-entrance of bacteria and diffusion of bacterial products from root canal system into the periapical tissues and vice versa [3-5]. 

The ideal material for root-end filling should be biocompatible, dimensionally stable, insoluble and most importantly, should be able to seal the root canal system [1, 6]. In addition, the handling properties are critical for root-end filling materials [6, 7]. In endodontic surgery, many materials have been used for retrofilling, such as amalgam, super-EBA, composite resin and glass ionomer [6-8]. However, most of them exhibit significant shortcomings in one or more properties such as leakage, solubility, biocompatibility, handling properties and moisture incompatibility [7]. 

Mineral trioxide aggregate (MTA) was developed at Loma Linda University in the early 1990s [9] and its principal components are tricalcium silicate, tricalcium aluminate, tricalcium oxide and silicon oxide [1, 9]. MTA has much more advantages over other root-end filling materials in terms of sealing ability [1, 4, 10, 11], marginal adaptation [11, 12] and biocompatibility [6, 13]. MTA has been commonly used as a preferred root-end filling material. However, MTA is not entirely accepted because of some drawbacks including the relatively long setting time (approximately 4 h), handling difficulty because of the initial looseness after mixing [5, 14] and the difficulty in maintaining the consistency of mixture [8].

Bioceramics are homogenous materials consisting of nanosphere particles, with the maximum particle dimension of 1×10^-3^ µm that are composed of tricalcium silicate, dicalcium silicate, calcium phosphate monobasic, amorphous silicon dioxide and tantalum pentoxide [15]. Novel bioceramic based materials were introduced in the form of root and perforation repair materials or sealers [15-18]. Among these, iRoot FS has been developed by Innovative BioCeramix Inc. (Vancouver, British Columbia, Canada). According to the manufacturer, iRoot FS is a ready-to-use premixed putty developed for permanent root canal repair and surgical applications. iRoot FS sets in the moist environment and does not shrink during the setting process, while maintaining excellent physical properties [19]. In an *in vitro* study, iRoot FS demonstrated negligible cytotoxicity, enhanced cell adhesion capacity and rapid setting [16, 20]. However, very few studies have been published regarding the sealing ability of iRoot FS as a root-end filling material in comparison with MTA.

**Figure 1:**
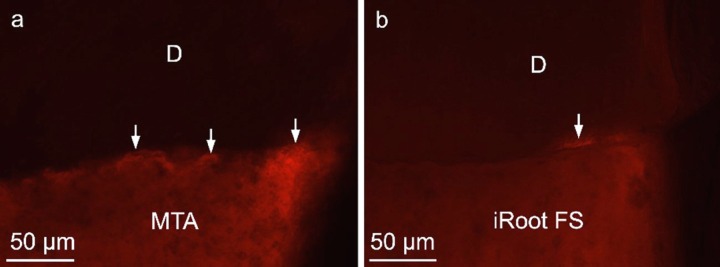
Fluorescent images of the longitudinal sections of the resected root-ends with retrofillings; *A)* MTA; and *B)* ) iRoot FS. Fluorescent coloring (white arrows) can be seen at the junction of the test material and dentin (D=dentine) (200× magnification)

The purpose of this study was to evaluate the sealing ability of iRoot FS and MTA as root-end filling materials by dye leakage method under fluorescence microscope.

## Materials and Methods

Forty single-canal human premolars extracted for orthodontic reasons were used in this study. Teeth with root caries, root fractures, significant apical curvatures, calcification, or resorption were excluded. Immediately after extraction, teeth were stored in 10% formalin solution until the experiment. 

***Root canal preparation***

After scaling and removing the remaining periodontal ligament, crowns were sectioned transversally at cemento-enamel junction with a diamond saw under continuous water spray. After that, the canals were instrumented using crown-down technique with ProTaper rotary instruments (Dentsply Maillefer, Ballaigues, Switzerland) with F3 (30/0.09) as the last apical file. Canals were irrigated with 5.25% sodium hypochlorite between the files. 

Then 5 mL of sterile saline was used as the final irrigant. After drying with absorbable paper points (Dayading, Beijing, China), the canals were filled with gutta-percha (Dentsply, Tianjin, China) and zinc oxide eugenol sealer (Produits Dentaires Pierre Rolland SAS, Merignac, France). The roots were then wrapped in moist gauze and stored in glass bottles for one week to prevent possible fracture during the cutting process [1].

**Figure 2:**
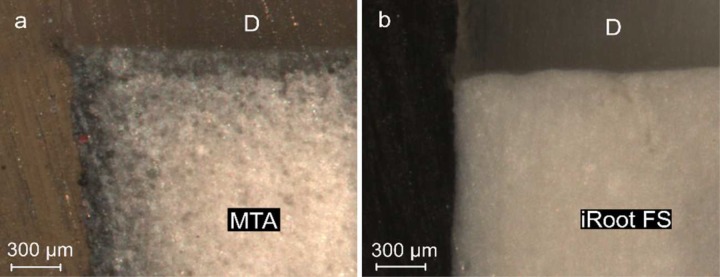
Longitudinal sections; *A)* The granular structure of MTA is clear. Dark discoloration is obvious on the surface and interface that gradually fades toward inside, *B)* iRoot FS; fine and smooth particles without any discoloration. (D=dentine) (63× magnification)

***Root-end preparation and filling***

By means of a diamond saw under continuous water spray three millimeter of root-ends were resected perpendicular to the long axis of the teeth [1, 11, 12]. Then 3 mm-deep root-end cavities were prepared using a diamond-coated ultrasonic surgical tip S12/90ND (Satelec/Acteon, Merignac, France). The integrity of the apical third was determined using a stereomicroscope under 32× magnification. The prepared roots were irrigated with 5 mL of 17% EDTA solution (Beyotine, Haimen, China) for 1 min to remove the smear layer, and then the canals were washed with distilled water for 5 min [21].

After preparation, the teeth were randomly divided into two experimental groups (*n*=18) and two control groups (*n*=2). The root-end cavities were rinsed with sterile saline and dried with paper points. In group A, tooth colored ProRoot MTA (Densply Tulsa Dental, Tulsa, OK, USA) was mixed according to the manufacturer’s instructions and condensed into the root-end cavities using #2 hand pluggers (Dentsply Maillefer, Ballaigues, Switzerland). The excess material on the root surfaces was cleaned with moist gauze. In group B, the root-end cavities were filled with iRoot FS (Innovative BioCeramix, Vancouver, BC, Canada) directly from the container with ready to use, factory pre-mixed putty. The material was also condensed into the cavities using #2 hand pluggers. The excess material on the root surfaces was cleaned with moist gauze. In the positive control group, root-end cavities were prepared in two teeth and were left unfilled. In the negative control group the apical root preparations were filled with the test materials (MTA and iRoot FS). 

Radiographies were taken in mesiodistal and buccolingual directions to evaluate the root-end fillings. The surfaces of the teeth in groups A, B and the positive control group were double coated with nail polish except for the apical area that was submitted to apicectomy [6, 10, 11]. The entire external surfaces of the teeth in negative control group were double coated with nail polish including the apex. All roots were wrapped in wet gauze and stored in 100% humidity for 24 h.

**Figure 3:**
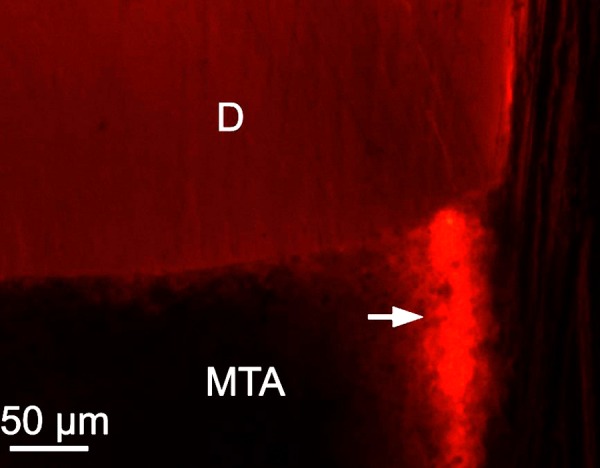
Fluorescent images of the longitudinal sections of the specimen filled with MTA. Fluorescent coloring stripe (white arrow) is clear on the surface of MTA (D=dentin) (100× magnification)

***Evaluation of dye penetration***

All roots were immersed in 0.2% Rhodamine B dye (Sigma-Aldrich, St. Louis, MO) so that the coronal portion was left out of the solution and were left at room temperature for 48 h. The roots were then immersed in tap water for 12 h and longitudinally sectioned into two halves using a 0.20 mm-thick diamond band saw (Exakt, Norderstedt, Germany) under continuous irrigation. When excited with green light of 550 nm, Rhodamine B exerts a red-orange fluorescence. The extent of dye penetration along the cavity was measured quantitatively in micrometers using a camera and monitored with the fluorescence microscope (Eclipse 80i, Nikon, Tokyo, Japan). Each specimen was measured independently by two observers at two different times under similar conditions. The average of the same measurement region from the two observers was recorded. For each sample, two mean values were double checked and recorded, one for each of the cavity walls in each half of the specimen and the other for the extent of dye penetration.

Also the discoloration of tooth structure induced by the root-end filling material was visually evaluated in both experimental groups.

***Statistical analysis***

Data were analyzed using SPSS software (SPSS version 13.0, SPSS, Chicago, IL, USA). The Mann-Whitney U test was used to determine the statistical difference of dye leakage between the two groups. The level of significance was set at 0.05.

## Results

Fluorescent coloring could be seen at the junction of test materials and dentin in both experimental groups (Figure 1). The mean leakage of MTA and iRoot FS were 35.63 and 35.15 μm, respectively. There was no significant difference between the leakage results for the two experimental groups (*P*=0.584). The positive control samples had significantly more leakage than the experimental groups. The negative controls showed no leakage.

The dark discoloration of tooth structure was observed in almost all of the MTA specimens. The discoloration was more obvious on the surface and the material-tooth interface and became gradually weak when moving towards the interior (Figure 2A). However discoloration was not observed in iRoot FS samples (Figure 2B). A continuous fluorescent coloring was observed on the surface of MTA in some specimens (Figure 3).

## Discussion

In this study, the apical sealing ability of iRoot FS and MTA root-end fillings was compared using dye penetration method. The mean values of leakage in MTA and iRoot FS samples were similar, and there was no significant difference between the two materials. However the dark discoloration of tooth structure was observed in almost all of the MTA specimens.

The quality of apical sealing ability obtained by various root canal repair and root-end filling materials can be assessed by different methods including dye penetration, radioisotope, bacterial penetration, electrochemical means, scanning electron microscope (SEM) examination and fluid filtration technique [1, 4, 5]. 

A dye penetrant model was chosen for this study because it is the easiest and the most widely-used method to screen the leakage of new restorative filling materials [1, 3, 22]. Bioceramics are bioavailable ceramic materials specifically designed for use in medicine and dentistry. Bioceramics possess excellent chemical durability, wear resistance, biocompatibility, environmental friendliness and aesthetics which can be contributed to their nano-sized particles [23]. MTA has presented good sealing ability in several studies [1, 4, 10, 11]. Therefore MTA was used as a gold standard for comparison. However, there have been some disadvantages with clinical application of MTA in terms of the relatively long setting time (approximately 4 h), difficulty to manipulate [5, 14] and dark discoloration of the tooth [24-26].

In the present study, we have also observed similar discoloration phenomenon in the MTA samples. Steffen *et al.* [27] consider the bismuth oxide component (added for better radiopacity) responsible for the dark discoloration of MTA. Interestingly, the dark discoloration was not observed in the iRoot FS samples (which is bismuth-free). This observation seems to confirm the linkage of the discoloration to the presence of bismuth. When used as a pulp capping agent in vital pulp therapy, tooth discoloration caused by MTA will affect the aesthetics, especially in anterior teeth. Thus it may be more appropriate to use iRoot FS particularly in the aesthetic zone [15]. 

In this study, iRoot FS showed similar extent of dye penetration compared to MTA. The explanation for the excellent sealing ability might be that iRoot FS does not shrink during the setting process [19]. Similarly, the variability of the sealing performance of iRoot FS was less than that of MTA. The greater range of measured values in MTA samples could be caused by the insufficient and/or non-homogeneous mixing of MTA [28, 29]. It puts an emphasis on the fact that the characteristics of MTA were simultaneously affected by multiple difficult-to-control factors. However, as a premixed bioceramic, iRoot FS delivered a consistent homogeneous product in every application, which could be contributed to the much more concentrated penetration values measured in this experiment. 

Fluorescent coloring on the surface was observed in some of the specimens. It can be said that the setting properties of MTA might be contributed to this phenomenon. The manufacturer recommends mixing MTA with sterile water, and this produces a grainy, sandy mixture with rather poor consistency [30, 31]. The residual porosity of MTA caused by the excess water (*i.e.* water not consumed in the hydration reaction) while setting and/or entrapped air might provide a space for dye penetration [32, 33]. It must be noted that the excess water improves flowability of MTA and thus the user may tempt to use more water, triggering higher porosity and thus larger flaws in the set material. Flaws in the set material might aggravate the cracks which inevitably exist as a result of the stresses developing during initial setting [32]. It has also been demonstrated that the addition of bismuth oxide decreased the mechanical stability by introducing more flaws and increased porosity by leaving more unreacted water within MTA [32, 34, 35]. Compared with MTA, the particle size of iRoot FS is much finer, which can be seen in the "feature-less" microstructure shown in Figure 2B. It was observed that the finer particles and dimensional stability during setting made it possible for iRoot FS to better fit the microscopic features and structure of a root canal wall and thus lead to better sealing ability [18, 36].

Handling characteristics are important when considering any material for clinical use [37]. MTA is a powder consisting of hydrophilic fine particles and sets in the presence of water [1]. The physical characteristics of MTA depend on the particle size, setting temperature, the powder to water ratio and entrapped air during mixing [9]. Some of these factors are difficult to control and others may not be at the optimum level after combining MTA with water in the clinical environment. It is therefore stated that the mixing of MTA is usually insufficient and lead to non-homogeneous distribution of the powder in water [28, 29]. Premixed bioceramic root filling materials offer the advantages of shortened application time and added application convenience, which is particularly important in clinical environment. Paste bioceramics deliver a consistent (factory pre-mixed) homogeneous product into the target point which can reduce the waste of material and increase utilization by taking only the required amount of the material [16]. In addition, the setting time of iRoot FS is about 30 min. Similar to MTA, iRoot FS also requires the presence of water to set and harden which is conveniently supplied partially through the material contact with tissue and body fluids, and partially through the *in situ* reactions within the material [17]. 

In summary, it appears that iRoot FS has overcome the disadvantages of MTA regarding poor handling, product variability through changing preparation (mixing) conditions and the long setting time. As the present study investigated the sealing properties under perfect conditions, it is advisable to consider these favorable results within the hostile oral environment clinical situations.

## Conclusion

In this study iRoot FS and MTA showed similar sealing ability, although iRoot FS had significantly less scatter compared to MTA.
